# Effectiveness of Ad26.COV2.S Vaccine vs BNT162b2 Vaccine for COVID-19 Hospitalizations

**DOI:** 10.1001/jamanetworkopen.2022.0868

**Published:** 2022-03-02

**Authors:** Jérémie Botton, Laura Semenzato, Marie-Joëlle Jabagi, Bérangère Baricault, Alain Weill, Rosemary Dray-Spira, Mahmoud Zureik

**Affiliations:** 1 EPI-PHARE Scientific Interest Group in Epidemiology of Health Products, French National Agency for the Safety of Medicines and Health Products, French National Health Insurance, Saint-Denis, France

## Abstract

This comparative effectiveness research study examines the effectiveness of full vaccination with Ad26.COV2.S vs BNT162b2 against COVID-19–related hospitalization.

## Introduction

Although the Ad26.COV2.S vaccine (Janssen) showed an efficacy of 85.4% against severe and critical COVID-19 in the pivotal trial,^1^ its effectiveness in the general population against COVID-19 hospitalization was estimated to be approximately 68%,^2,3^ compared with approximately 90% for mRNA vaccines.^4^ However, to date, the effectiveness of Ad26.COV2.S has not been compared with that of other COVID-19 vaccines. In France, the Ad26.COV2.S vaccine was used from April 24, 2021, in people aged 55 years or older, whereas the BNT162b2 mRNA vaccine (Pfizer-BioNTech) was the most widely administered (78% of first doses). As of the end of July 2021, 19 million people aged 55 years or older (84% of the population in that age group) were partially or fully vaccinated. In this comparative effectiveness research study, we compare the effectiveness of full vaccination with Ad26.COV2.S vs BNT162b2 against COVID-19–related hospitalization.

## Methods

The research group has permanent regulatory access to the anonymized data from the French National Health Data System (French decree No. 2016-1871, French law articles Art. R. 1461-13/14, French data protection authority decision CNIL-2016-316). Thus, no informed consent or specific approval by an ethics committee was required. This report follows the International Society for Pharmacoeconomics and Outcomes Research (ISPOR) reporting guideline for comparative effectiveness research.

On the basis of the French National Health Data System^5^ (see the eAppendix in the Supplement), we constructed a matched cohort of participants aged 55 years or older vaccinated with either Ad26.COV2.S or BNT162b2 between April 24, 2021, and July 31, 2021 (ie, 99 days). Participants of the 2 groups were individually matched according to age, sex, area of residence (100 areas) and date of full vaccination (first dose for Ad26.COV2.S, second dose for BNT162b2). Each participant was followed from the date of injection (day 0) or day 14 or day 28 (time of full effectiveness) until hospitalization for COVID-19 (outcome), death, or the end of follow-up on August 31, 2021, whichever occurred first.

The COVID-19 hospitalization rate was compared between the 2 groups using inverse probability of treatment weighted Cox models. Data were analyzed using SAS statistical software version 9.4 (SAS Institute).

## Results

The cohort included 689 275 participants vaccinated with Ad26.COV2.S (94% of all individuals of this age category vaccinated with Ad26.COV2.S) and 689 275 participants vaccinated with BNT162b2. The mean (SD) age was 65.8 (9.0) years, and 341 490 participants in each group (49.5%) were women. The 2 groups were similar in terms of socioeconomic and health characteristics (Table 1). During a median (IQR) follow-up of 54 (22-74) days from day 28 after injection, 129 COVID-19–related hospitalizations occurred in participants vaccinated with Ad26.COV2.S, and 23 hospitalizations occurred in those vaccinated with BNT162b2. The risk of hospitalization for COVID-19 from day 28 after injection was 5.2 times higher in individuals vaccinated with Ad26.COV2.S compared with those vaccinated with BNT162b2 (adjusted hazard ratio, 5.2; 95% CI, 3.4-7.9) (Table 2). On the basis of these results and according to an effectiveness of BNT162b2 of 92% (95% CI, 90%-94%) estimated from the same data set,^6^ we obtained an absolute effectiveness of Ad26.COV2.S of 59% (95% CI, 33%-75%).

**Table 1.** Characteristics of the Study Population at the Index Date, by Vaccine Received

Baseline characteristics	Participants, No. (%)		Standardized difference
	Pfizer (n = 689 275)	Janssen (n = 689 275)	
Age, mean (SD), y	65.8 (9.0)	65.8 (9.0)	0.00
Age range, y			
55-59	203 666 (29.5)	203 666 (29.5)	0.00
60-64	165 888 (24.1)	165 888 (24.1)	
65-69	129 310 (18.8)	129 310 (18.8)	
70-74	85 465 (12.4)	85 465 (12.4)	
75-79	38 676 (5.6)	38 676 (5.6)	
80-84	27 089 (3.9)	27 089 (3.9)	
85-89	22 894 (3.3)	22 894 (3.3)	
≥90	16 287 (2.4)	16 287 (2.4)	
Sex			
Female	341 490 (49.5)	341 490 (49.5)	0.00
Male	347 785 (50.5)	347 785 (50.5)	
Administrative regions			
Auvergne-Rhône Alpes	70 286 (10.2)	70 286 (10.2)	0.00
Bourgogne Franche Comté	31 072 (4.5)	31 072 (4.5)	
Bretagne	46 299 (6.7)	46 299 (6.7)	
Centre-Val de Loire	33 181 (4.8)	33 181 (4.8)	
Corse	1322 (0.2)	1322 (0.2)	
Grand Est	57 914 (8.4)	57 914 (8.4)	
Guadeloupe	121 (<0.1)	121 (<0.1)	
Guyane	29 (<0.1)	29 (<0.1)	
Hauts de France	67 759 (9.8)	67 759 (9.8)	
Ile de France	77 758 (11.3)	77 758 (11.3)	
La Réunion	9816 (1.4)	9816 (1.4)	
Martinique	602 (0.1)	602 (0.1)	
Mayotte	10 (<0.1)	10 (<0.1)	
Normandie	42 934 (6.2)	42 934 (6.2)	
Nouvelle Aquitaine	85 172 (12.4)	85 172 (12.4)	
Occitanie	68 557 (9.9)	68 557 (9.9)	
Pays de Loire	46 019 (6.7)	46 019 (6.7)	
Provence Alpes Cote	50 424 (7.3)	50 424 (7.3)	
Index of deprivation (quintiles)			
1 (less deprived)	111 954 (16.2)	103 143 (15.0)	0.05
2	130 654 (19.0)	125 758 (18.2)	
3	140 763 (20.4)	141 421 (20.5)	
4	149 910 (21.7)	154 981 (22.5)	
5 (more deprived)	143 485 (20.8)	151 430 (22.0)	
Unknown	12 509 (1.8)	12 542 (1.8)	
Influenza vaccination in 2018 or 2019			
No	575 478 (83.5)	595 934 (86.5)	−0.08
Yes	113 797 (16.5)	93 341 (13.5)	
Frailty			
No	655 054 (95.0)	647 004 (93.9)	0.05
Yes	34 221 (5.0)	42 271 (6.1)	
Addiction to alcohol			
No	678 987 (98.5)	670 557 (97.3)	0.09
Yes	10 288 (1.5)	18 718 (2.7)	
Addiction to tobacco smoking			
No	652 205 (94.6)	644 552 (93.5)	0.05
Yes	37 070 (5.4)	44 723 (6.5)	
Hypertension			
No	431 663 (62.6)	434 557 (63.0)	−0.01
Yes	257 612 (37.4)	254 718 (37.0)	
Diabetes			
No	611 466 (88.7)	604 658 (87.7)	0.03
Yes	77 809 (11.3)	84 617 (12.3)	
Dyslipidemia			
No	539 301 (78.2)	546 973 (79.4)	−0.03
Yes	149 974 (21.8)	142 302 (20.6)	
Obesity			
No	678 705 (98.5)	678 452 (98.4)	0.00
Yes	10 570 (1.5)	10 823 (1.6)	
Coronary diseases			
No	646 997 (93.9)	651 528 (94.5)	−0.03
Yes	42 278 (6.1)	37 747 (5.5)	
Heart failure			
No	677 681 (98.3)	676 259 (98.1)	0.02
Yes	11 594 (1.7)	13 016 (1.9)	
Cardiac rhythm or conduction disturbances			
No	662 974 (96.2)	664 891 (96.5)	−0.01
Yes	26 301 (3.8)	24 384 (3.5)	
Valvular diseases			
No	678 088 (98.4)	679 521 (98.6)	−0.02
Yes	11 187 (1.6)	9754 (1.4)	
Obliterating arterial disease of the lower limb			
No	676 328 (98.1)	674 289 (97.8)	0.02
Yes	12 947 (1.9)	14 986 (2.2)	
Stroke			
No	673 133 (97.7)	673 422 (97.7)	0.00
Yes	16 142 (2.3)	15 853 (2.3)	
Pulmonary embolism			
No	686 537 (99.6)	687 242 (99.7)	−0.02
Yes	2738 (0.4)	2033 (0.3)	
Chronic respiratory diseases (excluding cystic fibrosis)			
No	643 123 (93.3)	641 582 (93.1)	0.01
Yes	46 152 (6.7)	47 693 (6.9)	
Long-term dialysis			
No	688 913 (99.9)	689 163 (100)	−0.02
Yes	362 (0.1)	112 (<0.1)	
Kidney transplant			
No	688 626 (99.9)	689 192 (100)	−0.04
Yes	649 (0.1)	83 (<0.1)	
Liver diseases			
No	682 503 (99.0)	681 875 (98.9)	0.01
Yes	6772 (1.0)	7400 (1.1)	
Active cancers			
No	667 567 (96.9)	674 000 (97.8)	−0.06
Yes	21 708 (3.1)	15 275 (2.2)	
Neurotic and mood disorders, use of antidepressant treatments			
No	617 809 (89.6)	610 159 (88.5)	0.04
Yes	71 466 (10.4)	79 116 (11.5)	
Psychotic disorders, use of neuroleptics treatments			
No	680 647 (98.7)	675 353 (98.0)	0.06
Yes	8628 (1.3)	13 922 (2.0)	
Dementias (including Alzheimer disease)			
No	683 401 (99.1)	682 000 (98.9)	0.02
Yes	5874 (0.9)	7275 (1.1)	
Epilepsy			
No	685 812 (99.5)	685 027 (99.4)	0.02
Yes	3463 (0.5)	4248 (0.6)	
Parkinson disease			
No	683 236 (99.1)	681 850 (98.9)	0.02
Yes	6039 (0.9)	7425 (1.1)	
Chronic inflammatory bowel diseases			
No	685 482 (99.4)	686 773 (99.6)	−0.03
Yes	3793 (0.6)	2502 (0.4)	
Rheumatoid arthritis and related diseases			
No	683 125 (99.1)	685 008 (99.4)	−0.03
Yes	6150 (0.9)	4267 (0.6)	
Ankylosing spondylitis and related diseases			
No	685 396 (99.4)	686 689 (99.6)	−0.03
Yes	3879 (0.6)	2586 (0.4)	

**Table 2.** Comparison of Ad26.COV2.S and BNT162b2 Vaccines in Terms of Risk of Hospitalization for COVID-19 in France

Follow-up intervals and vaccine	Participants, No.	Events, No. (%)	Follow-up, median (IQR), d	HR (95% CI)^a^	
				Crude	Adjusted
Day 0 to the end of follow-up					
BNT162b2	689 275	49 (0.01)	82 (50-101)	1 [Reference]	1 [Reference]
Ad26.COV2.S	689 275	285 (0.04)	82 (49-101)	5.82 (4.30-7.88)	5.51 (4.11-7.40)
Day 14 to the end of follow-up					
BNT162b2	688 861	28 (0.00)	68 (36-88)	1 [Reference]	1 [Reference]
Ad26.COV2.S	688 861	203 (0.03)	68 (36-87)	7.26 (4.89-10.77)	6.70 (4.59-9.75)
Day 28 to the end of follow-up					
BNT162b2	688 263	23 (0.00)	54 (22-74)	1 [Reference]	1 [Reference]
Ad26.COV2.S	688 263	129 (0.02)	54 (22-74)	5.61 (3.60-8.75)	5.16 (3.39-7.85)

Abbreviation: HR, hazard ratio.

^a^ HRs were obtained from inverse probability of treatment weighted Cox models taking into account all the variables described in Table 1.

## Discussion

This comparative effectiveness research study, which, to our knowledge, is the largest estimating effectiveness of Ad26.COV2.S in the general population, included almost the entire population aged 55 years or older vaccinated with Ad26.COV2.S in France. Considering the high rate of vaccination uptake in this population, using an active comparator was more relevant than considering unvaccinated individuals as controls. The risk of severe COVID–19 related hospitalization after vaccination was approximately 5 times higher with Ad26.COV2.S than with BNT162b2.

On the basis of these results and an effectiveness of BNT162b2 of 92% (95% CI, 90%-94%) estimated from the same data set,^6^ we obtained an absolute effectiveness of Ad26.COV2.S of 59% (95% CI, 33%-75%). This finding is consistent with previous estimates of smaller populations and using test-negative or case-control designs.^2,3^ A limitation of this study is that, although the 2 vaccine groups were matched on vaccination day, age, sex, and area of residence and the associations were adjusted for a large number of covariables, we cannot completely exclude residual confounding. Using the active BNT162b2 vaccine comparator likely lowered this potential bias compared with using an unvaccinated group. The slightly higher risk estimates from day 0 or 14 are likely associated with partial protection by the first dose of BNT162b2 combined with delayed protection of Ad26.COV2.S immediately in the days after injection.

## Conclusions

This study found that the Ad26.COV2.S vaccine is less effective against COVID-19–related hospitalization than the BNT162b2 vaccine. These results strengthen the evidence supporting a second dose in people who received the Ad26.COV2.S vaccine by an mRNA vaccine as recommended in both France and the US.

## Notes

Supplement. **eAppendix.** Supplemental Methods
eReferences
