# Supplementary material for: Effectiveness of Ad26.COV2.S Vaccine vs BNT162b2 Vaccine for COVID-19 Hospitalizations
Source: JAMA Netw Open. 2022 Mar 2;5(3):e220868. doi: 10.1001/jamanetworkopen.2022.0868 (PMC8892225; doi:10.1001/jamanetworkopen.2022.0868)
Supplement: Supplement. — eAppendix. Supplemental Methods eReferences [file jamanetwopen-e220868-s001.pdf]

Supplemental Online Content

Botton J, Semenzato L, Jabagi MJ, et al. Effectiveness of Ad26.COV2.S vaccine vs BNT162b2 vaccine for COVID-19 hospitalizations. *JAMA Netw Open*. 2022;5(3):e220868.
doi:10.1001/jamanetworkopen.2022.0868

eAppendix. Supplemental Methods

eReferences

This supplemental material has been provided by the authors to give readers additional information about their work.

© 2022 Botton J et al. *JAMA Network Open.*

eAppendix. Supplemental Methods

Data sources: the French National Health Data System SNDS

This study was based on the National Health Data System (SNDS) that covers the entire French population, i.e. 67 million inhabitants, and which has been extensively used in France to conduct pharmaco-epidemiology studies including studies on the COVID-19 pandemic (References below).

Since 2006, a unique, anonymous individual identifier links data derived from two main databases: the DCIR (Datamart de Consommation Inter-Regimes, the national health insurance reimbursement database) and the PMSI (Programme de médicalisation des Systèmes d'Information, the national hospital database). Each patient is identified by a unique identifier (encrypted twice in the SNDS) for data collection. The INSEE number (INSEE: Institut National de la Statistique et des Études Économiques - French National Institute for Statistics and Economic Studies) is a combination of 13 figures attributed to each French person at birth or at the time of legal immigration of any person residing in France. INSEE is responsible for assigning this registration number at the time of civil status registration. All adults legally residing in France have a national health insurance card ("Carte Vitale"), equipped with an electronic chip. When dispensing a drug, the pharmacist scans the bar code on the drug package (brand name, dose strength, number of tablets) and records the patient's identifier by means of the Carte Vitale. Patients must also present their Carte Vitale whenever they are admitted to hospital, as funding of the patient's hospital stay and treatment outside of hospital is dependent on the patient's identifier.

The DCIR includes individual data concerning reimbursements of outpatient medical care, laboratory tests and drugs, coded according to the Anatomical Therapeutic Chemical (ATC) classification. Health expenditure for people with long-term diseases (ALDs), such as cancer or diabetes, is fully covered financially and their diagnoses are registered according to the International Classification of Diseases, 10th Revision (ICD-10).

The PMSI indicates the dates of admission and discharge for all public or private hospital stays in France. Medical diagnoses are coded according to the ICD-10 classification and the main medical or surgical procedures are coded according to the Classification Commune des Actes Médicaux (CCAM).

© 2022 Botton J et al. *JAMA Network Open.*

Patients receiving at least one health care reimbursement after 1 January 2018 were identified from the SNDS and were considered to be included in this study. Twins and foreign residents were excluded due to identification difficulties, and we also excluded people with missing data for age and sex or who died before 24 April 2021.

Description of the variables

We considered the patient's age, gender and region of residence as matching variables. Age was defined as a categorical variable by 5-year-age groups. We used the social deprivation index as a measure of socioeconomic status. This indicator is based on the median household income, the percentage of high school graduates in the population over the age of 15, the percentage of manual workers in the labour force and the unemployment rate for the person's town of residence.

The mapping of diseases and expenditure is a tool developed from the DCIR and PMSI databases, allowing identification of diseases by means of medical algorithms (47 diseases were studied in the present study) based on the reasons for hospitalization, ALD diagnoses and/or reimbursement of specific treatments for certain diseases, over a period of the previous 4 years. The detailed definition of these disease identification algorithms is publicly available in French (https://www.ameli.fr/fileadmin/user_upload/documents/Methodologie_medicale_cartographie.pdf). Mapping algorithms allowed the identification of patients presenting a number of different diseases in 2020 and were completed by the identification of obese patients, smokers and people with alcohol use disorder. The following chronic diseases were considered: cardiometabolic diseases, such as obesity, diabetes, hypertension, dyslipidaemia and/or lipid-lowering drug treatment or cardiovascular diseases (stroke and stroke sequelae, heart failure, coronary heart disease, cardiac arrhythmias or conduction disorders, valvular heart disease, peripheral artery disease), chronic respiratory diseases (excluding cystic fibrosis), pulmonary embolism, female breast, lung, prostate, colorectal cancer and other active cancers, inflammatory diseases or skin diseases (chronic inflammatory bowel disease [IBD], rheumatoid arthritis, ankylosing spondylitis and related diseases, psoriasis), mental and behavioural disorders, neurodegenerative diseases and severe chronic kidney disease.

© 2022 Botton J et al. *JAMA Network Open.*

Design and statistical analyses

We constructed a cohort of 55 years and older subjects vaccinated with the Janssen vaccine between 24 April 2021 and 31 July 2021 (i.e. 99 days). Each subject was matched to a subject vaccinated with the Pfizer / BioNTech vaccine of the same age, sex, administrative region and date of injection (first dose for Janssen and second dose for Pfizer / BioNTech vaccine). Pairs with at least one of the participant with prior COVID-19 hospitalization were excluded (5%).The pairs were followed from this index date.

The endpoint of interest was hospitalization for COVID-19 during follow-up. Each subject was followed from the index date until hospitalization for COVID-19, death, or the end of follow-up on 31 August 2021, whichever occurred first. Different time windows were considered: from injection until the end of follow-up, from day 14 after injection until the end of follow-up and from day 28 after injection until the end of follow-up.

Characteristics of the two cohorts were compared (see Table 1 in the main text). The COVID-19 hospitalization rate in vaccinated subjects with Janssen vaccine was compared to those among vaccinated subjects with the Pfizer / BioNTech vaccine by Cox models taking into account individual comorbidities by using of propensity score method, by inverse probability of treatment weighting. Adjusting Cox-model for all the covariables gave similar results.

The own-effectiveness of Janssen vaccine was calculated by first calculating the product of Hazard Ratio (HR) of Janssen (J) compared to Pfizer and the HR of Pfizer (P) compared to unvaccinated people (0) obtained from our previous study:

$$HR_{j/0} = IR_j / IR_0 = IR_j / IR_p * IR_p / IR_0 = HR_{j/p} * HR_{p/0}$$

To estimate confidence intervals of this HR j/0, we first calculated the variance of log(HR j/0) as the sum of the variances of the coefficients $beta_{j/p}$ and $beta_{p/0}$, the logarithms of corresponding HR. Then, confidence limits were obtained as the exponentials of beta +/- 1.96 $sqrt(var(log(HR_{j/0})))$.

Risk reduction was finally defined as the percent reduction in risk, calculated as 1 minus the $HR_{j/0}$.

© 2022 Botton J et al. *JAMA Network Open.*

eReferences

1. Bouillon K, Bertrand M, Bader G, Lucot J-P, Dray-Spira R, Zureik M. Association of Hysteroscopic vs Laparoscopic Sterilization With Procedural, Gynecological, and Medical Outcomes. JAMA. 2018;319(4):375-387. doi:10.1001/jama.2017.21269
2. Weill A, Dalichampt M, Raguideau F, Ricordeau P, Blotière P-O, Rudant J, Alla F, Zureik M. Low dose oestrogen combined oral contraception and risk of pulmonary embolism, stroke, and myocardial infarction in five million French women: cohort study. BMJ. 2016;353:i2002. doi:10.1136/bmj.i2002
3. Maura G, Blotière P-O, Bouillon K, Billionnet C, Ricordeau P, Alla F, Zureik M. Comparison of the short-term risk of bleeding and arterial thromboembolic events in nonvalvular atrial fibrillation patients newly treated with dabigatran or rivaroxaban versus vitamin K antagonists: a French nationwide propensity-matched cohort study. Circulation. 2015;132(13):1252-1260. doi:10.1161/CIRCULATIONAHA.115.015710
4. Meyer A, Rudant J, Drouin J, Weill A, Carbonnel F, Coste J. Effectiveness and Safety of Reference Infliximab and Biosimilar in Crohn Disease: A French Equivalence Study. Ann Intern Med. 2019;170(2):99-107. doi:10.7326/M18-1512
5. Tubiana S, Blotière P-O, Hoen B, Lesclous P, Millot S, Rudant J, Weill A, Coste J, Alla F, Duval X. Dental procedures, antibiotic prophylaxis, and endocarditis among people with prosthetic heart valves: nationwide population based cohort and a case crossover study. BMJ. 2017;358:j3776.
6. Rachas A, Gastaldi-Menager C, Denis P, Lesuffleur T, Nicolas M, Pestel L, Mette C, Drouin J, Riviere S, Tajahmady A, Gissot C, Fagot-Campagna A. Prevalences and healthcare expenditures related to 58 health conditions from 2012 to 2017 in France: diseases and healthcare expenditure mapping, a national population-based study. medRxiv. Published online January 1, 2020:2020.09.21.20198853. doi:10.1101/2020.09.21.20198853
7. Semenzato L, Botton J, Drouin J, Cuenot F, Dray-Spira R, Weill A, Zureik M. Chronic diseases, health conditions and risk of COVID-19-related hospitalization and in-hospital mortality during the first wave of the epidemic in France: a cohort study of 66 million people. Lancet Reg Health Eur. 2021 Sep;8:100158. doi: 10.1016/j.lanepe.2021.100158.

© 2022 Botton J et al. *JAMA Network Open.*
